# The Treatment of Pneumonia, with Special Reference to Creosote*Read before the Brazos Valley Medical Association at the eleventh semiannual meeting at Calvert, Texas, May 14, 1901.

**Published:** 1901-12

**Authors:** H. W. Cummings

**Affiliations:** Hearne, Texas


### TEXAS MEDICAL JOURNAL.

ESTABLISHED JULY, 1885.

PUBLISHED MONTHLY.—SUBSCRIPTION $1.00 A YEAR.

VOL. XVII. AUSTIN, DECEMBER, 1901. No. 6.

### Original Contributions.

For Texas Medical Journal.

### The Treatment of Pneumonia, With Special Reference to Creosote.*

BY H. W. CUMMINGS, M. D., HEARNE, TEXAS.

It is not my purpose to write a paper upon the pathology and symptoms of pneumonia, nor to deal in a general way with its treatment; but, in as brief a manner as possible, to relate to you some results I have had from the use of creosote in the management of this disease.

In January last, I listened with much interest to a paper read before the Central Texas Medical Association by Dr. I. L. Van Zandt, of Fort Worth, Texas. His subject was "Creosote in Pneumonia." The paper was a resumè of seven years' experience, and he cited a number of cases with their history and symptoms which had been treated by the use of creosote. According to his assertions he had been able to limit the duration of pneumonia to three or four days, with a few exceptions, and in some instances as short as two to three days.

I concluded that if such was the experience of Dr. Van Zandt, I would try the treatment myself, and should I succeed as well as he,

*Read before the Brazos Valley Medical Association at the eleventh semi-annual meeting at Calvert, Texas, May 14, 1901.

I would be well paid for my investigations and my patients relieved of much suffering and anxiety.

Formerly I had treated pneumonia upon the expectant plan, meeting symptoms as they appeared, but constantly stimulating my patients with strychnine and supporting them with a light, nutritious diet.

Since January, I have treated twenty cases of pneumonia, using creosote in each, and out of the twenty cases I have had no deaths.

Of these twenty cases seven have reached the crisis in four days, seven in five days, two in six days, two in seven days, and one each in eight and nine days.

This shows fourteen cases out of twenty that reached the crisis by the fifth day, while one only lasted as long as the ninth day.

Now, while we are taught by some of the best authorities that pneumonia reaches its crisis between five and ten days, my former experience has been that all my cases of lobar pneumonia lasted from seven to ten days.

I also find that after I began the use of creosote the fever did not run as high as usual, nor did it fall so suddenly at the end of the disease, leaving the patient so prostrated, accompanied by the profuse sweat, but rather that the fever declined more gradually. Only one case showed any alarming symptoms at the crisis, and she had just recovered from a severe attack of la grippe when taken with pneumonia. Thus she was quite weak at the beginning.

Again, I find that the use of creosote lessens, to a great degree, the severity of the cough and pain, and my patients required but little medicine to produce rest or sleep. Not a single case ever vomited or suffered with nausea while the medicine was administered.

I do not make any special report of individual cases for the reason that I did not begin by keeping accurate report of each case, nor do I care here to discuss the general symptoms or treatment in this paper. I will state, however, that each case of these twenty presented the typical symptoms of pneumonia, and I have included none which were doubtful as to diagnosis.

You will note that six of these cases lasted from six to nine days, and it might be said that they were not influenced by the creosote, or else they also would have yielded in a shorter time.

In nearly every instance where the fever lasted beyond the fifth day it was in cases to which I was not called until about the second or third day after the chill. These, however, ran a much milder course, and rested better than was formerly the case in my practice.

We accept the theory that pneumonia is a specific disease, caused by a specific germ (the *dipplococci*), which manifests itself by a local inflammation in the lungs, having first the stage of congestion, then solidification or hepatization, after which, resolution. We further believe that these organisms are responsible for the general symptoms, especially the fever, by the intoxication of the system from this local manifestation.

Now we know that creosote is an antiseptic and germicide, and that taken into the system, it is eliminated unchanged through the kidneys, skin and lungs.

Accepting the above two propositions, is it not reasonable to believe that the elimination of this antiseptic through the lungs will, to some extent, at least, destroy these pneumococci or retard their development, thus reducing the amount of toxins absorbed into the system, lower the fever and cut short their destruction of lung tissue?

If given in time and in proper doses it is my opinion that you can shorten the duration and make milder an attack of pneumonia.

Now, as to the preparation I use, and the mode of administering it:

While you will probably get good results from the ordinary beech-wood creosote, I have used the carbonate of creosote, as suggested by Dr. Van Zandt, and find that it is more easily borne by the stomach and can be given in larger doses without any systemic disturbances.

I begin with ten drops every three hours, and increase the dose five drops daily until twenty to twenty-five drop doses are given for an adult. When the maximum dose is reached I place it at further intervals, say, four to five hours.

I might add that in all cases I use strychnine as a stimulent, beginning with small doses during the second stage and increase the dose as the patient approaches the crisis.

Now, gentlemen, I have not written this short sketch to show any particular originality, nor to claim any special ability in the treatment of pneumonia, but simply to give you in a few words the results I have had in the use of this remedial agent with the hope that you will consider it of sufficient value to make some trials of it, believing that you will find in it an agent of much value in the treatment of pneumonia as well as other diseases of the respiratory tract. I regret that I have not been able to give a more minute history of these cases, but I trust I have placed the subject before you with sufficient weight to elicit your discussion of the subject and your trial of the remedy.

In conclusion, it is my opinion that if we are able to cut short the duration of the disease, lessen the fever, allay the irritable cough and carry a larger per cent. of our patients to a safe convalescence the remedy is worthy your trial and investigation.

